# Phenotypic flexibility of energetics in acclimated Siberian hamsters has a narrower scope in winter than in summer

**DOI:** 10.1007/s00360-016-0959-3

**Published:** 2016-01-23

**Authors:** Jan S. Boratyński, Małgorzata Jefimow, Michał S. Wojciechowski

**Affiliations:** Department of Animal Physiology, Nicolaus Copernicus University, Toruń, Poland; Department of Vertebrate Zoology, Nicolaus Copernicus University, Toruń, Poland

**Keywords:** Phenotypic flexibility, Acclimation, Acclimatization, Thermoregulation, Energetics, Season

## Abstract

As photoperiod shortens with the approach of winter, small mammals should reduce their energy expenditure to survive periods of food limitation. However, within seasons, animals should balance their energy budgets as abiotic conditions change, sometimes unpredictably; cold spells should increase heat production, while warm spells should do the opposite. Therefore, we addressed specific questions about the possible interactions between seasonal acclimatization and the intra-seasonal phenotypic flexibility of metabolic rate. We hypothesized that phenotypic flexibility in small mammals differs seasonally and is greater in summer than in winter, and predicted that seasonal adjustments in energetics, which are driven by photoperiod, overwhelm the influence of variations in the thermal environment. We measured body mass, basal metabolic rate (BMR), facultative non-shivering thermogenesis (fNST), body temperature, and calculated minimum thermal conductance in Siberian hamsters *Phodopus sungorus*. Animals were acclimated to winter-like, and then to summer-like conditions and, within each season, were exposed twice, for 3 weeks to 10, 20 or 28 °C. We used differences between values measured after these short acclimation periods as a measure of the scope of phenotypic flexibility. After winter acclimation, hamsters were lighter, had lower whole animal BMR, higher fNST than in summer, and developed heterothermy. After these short acclimations to the above-mentioned temperatures, hamsters showed reversible changes in BMR and fNST; however, these traits were less flexible in winter than in summer. We conclude that seasonal acclimation affects hamster responses to intra-seasonal variations in the thermal environment. We argue that understanding seasonal changes in phenotypic flexibility is crucial for predicting the biological consequences of global climate changes.

**Electronic supplementary material:**

The online version of this article (doi:10.1007/s00360-016-0959-3) contains supplementary material, which is available to authorized users.

## Introduction

The phenotype is a product of genotype, environment and the interaction between them (Lynch and Walsh 1998). Depending on the environmental conditions during development, a single genotype can produce different phenotypes. This range of irreversibly expressed phenotypes is defined by developmental reaction norm (Platt and Sanislow 1988; Scheiner 1993; Pigliucci 2005). The same concept was adopted to define the scope of phenotypic flexibility, which represents reversible changes in behavior, morphology or physiology (McKechnie et al. 2007; McKechnie 2008; Nussey et al. 2007; Charmantier et al. 2008; van de Ven et al. 2013; Petit and Vézina 2014). These flexible adjustments can be caused by predictable (usually inter-seasonal) or unpredictable (usually intra-seasonal) changes in the environment (Piersma and Drent 2003). There are two general approaches to study these phenomena. One, testing the acclimatization of animals to seasonally changing environmental conditions (reviews in Lovegrove 2005; McKechnie 2008); the other, usually done under laboratory conditions, testing for physiological and behavioral responses to acclimations to specific environmental conditions (Li et al. 2001; Russell and Chappell 2007; McKechnie et al. 2007; Barceló et al. 2009; Chi and Wang 2011).

Seasonal changes in animal phenotypes are usually a response to predictable changes in photoperiod (Haim et al. 1995; Kronfeld-Schor et al. 2000; Bartness et al. 2002, Król et al. 2005) or ambient temperature (*T*_a_) (Rosenmann et al. 1975; Feist and Rosenmann 1976; Merritt and Zegers 1991; Condon et al. 2010) or both (Udaka et al. 2008). In winter, these seasonal changes lead to the development of an energy-conserving phenotype which is characterized by lower total energy expenditure, improved insulation and increased facultative heat production (Heldmaier 1989; Lovegrove 2005). Ultimately, these changes enable small mammals to survive periods of reduced energy availability and increased energy expenditure. In winter, many small mammals have decreased *m*_b_ and its greatest proportional decrease is observed in the smallest taxa (Heldmaier 1989; Lovegrove 2005). Although lower *m*_b_ should result in higher mass-specific metabolic rate (MR), the total energy expenditure of a smaller animal should be lower than in a larger animal (Heldmaier and Steinlechner 1981a, Bozinovic et al. 1990). Moreover, the larger the winter reduction of *m*_b_, the greater is the reduction of basal MR (BMR) in winter (Lovegrove 2005). Most small homeothermic mammals also change their pelage from summer to winter, which results in lower thermal conductance. On the one hand, the larger the winter reduction in thermal conductance, the greater is the reduction of BMR. On the other hand, the greater the increase in thermal conductance the greater is the seasonal reduction of body temperature (*T*_b_) (Lovegrove 2005). The reduction of *T*_b_ in winter also leads to a decrease in the body-to-ambient temperature difference, resulting in smaller heat loss and lower energy expenditure during normothermy (McNab 2002). Additionally, small mammals that are seasonally heterothermic develop the ability to enter torpor after several weeks of acclimation to winter-like conditions (Heldmaier and Steinlechner 1981b; Janský et al. 1984; Jefimow et al. 2004). Acclimation studies done under controlled conditions generally aim to test for the influence of particular factor (e.g., *T*_a_ or photoperiod) on animal phenotype. It was found that in most mammals and birds, changes in day length are the key environmental cues for seasonal changes in their physiology and life history traits (Heideman et al. 1999; Dawson et al. 2001; Bradshaw and Holzapfel 2007; Dawson 2007; Scherbarth and Steinlechner 2010). Seasonal changes in energetics were extensively studied in Siberian hamsters (*Phodopus sungorus*). During seasonal acclimatization this highly seasonal species relies on photoperiod (Wiesinger et al. 1989; Scherbarth and Steinlechner 2010) and develops a distinct winter phenotype characterized by decreased *m*_b_, molt to white pelage, regression of gonads, and development of the capacity to enter daily torpor (Heldmaier and Steinlechner 1981a; Jefimow et al. 2004).

Irrespective of season and corresponding photoperiod, animals may flexibly change their thermoregulatory mechanisms and energetics in response to intra-seasonal, sudden changes in *T*_a_ (Huey and Berrigan 1996). Thus, phenotypic flexibility may be considered an adaptation to living in unpredictable and variable environments (Piersma and Drent 2003). Sudden increase of energy requirements results in increased mass of the digestive organs (e.g., Derting and Bogue 1993; Bacigalupe et al. 2004; Russell and Chappell 2007), and eventually in increased BMR (Williams and Tieleman 2000; Nespolo et al. 2002; Klaassen et al. 2004; Vézina et al. 2006; McKechnie et al. 2007). Ultimately, it results in increased resting metabolic rate (RMR), altered enzyme activities, and increased capacity for non-shivering thermogenesis (NST; Nespolo et al. 1999) which, depending on species, may occur with or without changes in *m*_b_ (e.g., Li et al. 2001; Chi and Wang 2011).

Despite many studies of seasonal adjustments in animal energetics (for mammals: Heldmaier 1989; Lovegrove 2005; for birds: McKechnie 2008; Swanson 2010) as well as those examining physiological responses to short-term acclimations to controlled environments (Huey and Berrigan 1996), the interactions between physiological adjustments over these two timescales still remain elusive. In their recent study, Stager et al. (2015) found that over a quarter of the genes were differentially expressed under different thermal regimes in short and long photoperiods. Thus, it is justified to ask about the potential effect of the interaction between photoperiod and thermal conditions, namely whether seasonal acclimatization and corresponding changes in phenotype affect phenotypic flexibility in response to intra-seasonal exposure to different thermal conditions. To the best of our knowledge, so far there were no studies which would aim to answer this question using the same individuals which seasonally change their phenotype. Taking into account the postulated effects of the global climate change (IPCC 2007), it is also important to realize whether known patterns of phenotypic flexibility could be conservatively applied to animals acclimatized to different seasons. Animals living in highly seasonal environments rely primarily on day length as a signal for seasonal acclimatization and life cycle staging (Bradshaw and Holzapfel 2007), and thus would be more vulnerable to thermal perturbations in their environments than animals living in less seasonal environments (Canale and Henry 2010).

Pronounced phenotypic changes that occur in Siberian hamsters during acclimatization to winter give the opportunity to test the hypothesis that in small mammals short-term phenotypic flexibility differs between seasons, and is greater in summer than in winter. Such seasonal differences would be possible because seasonal changes of the phenotype are driven by hormones and are controlled by photoperiod (Heldmaier et al. 1990; Bartness et al. 1993; Prendergast 2010; Scherbarth and Steinlechner 2010). Thus, seasonal adjustments in energy expenditure, which are triggered by photoperiod, would overwhelm the effect of variations in thermal conditions on animal energetics. Specifically, with acclimation to winter-like conditions we predicted lower *m*_b_ and whole animal BMR, smaller thermal conductance (*C*), greater capacity for fNST and greater variability of *T*_b_. At the same time, after winter acclimation, we expected lower intra-seasonal changes of *m*_b_, BMR and fNST in response to changes of *T*_a_. Acclimation history as well as its duration and environmental conditions (here: temperature) may also affect longitudinal changes of *m*_b_, BMR, fNST and *C*, and their reversibility. According to its definition, phenotypic flexibility is reversible (Piersma and Drent 2003). Thus, we predicted that all traits will be reversible, irrespective of hamster acclimation history within each season. The time it takes for an individual to acclimate to given conditions may differ between individuals and one may acclimate faster than others (Rezende et al. 2004). Also, animals acclimated to a given L:D cycle, even under constant conditions, may change their physiology because of photorefractoriness (Masuda and Oishi 1995; Jefimow et al. 2005). Therefore, we maintained control groups under stable, seasonally specific *T*_a_ in both winter and summer photoperiod, and tested for possible longitudinal changes in the measured traits.

## Materials and methods

### Animals, housing and experimental design

The study was done at Nicolaus Copernicus University in Toruń, Poland between September 2012 and July 2013. All experimental procedures were approved by the Local Committee for Ethics in Animal Research in Bydgoszcz, Poland (decision number 19/2011). For the experiments, we randomly chose 40, 3-month-old male Siberian hamsters born in our breeding colony in summer 2012. We intentionally restricted the study to one sex to avoid potential effects of estrus cycle on hamster energetics and thermoregulation. Animals were kept singly in standard rodent cages (model number: 1246; Tecniplast, Italy) with wood shavings and access to food and water ad libitum. Hamsters were fed with standard rodent diet (Labofeed B, Morawski, Kcynia, Poland). During winter acclimation, we supplemented their diet once a week with sunflower seeds (8 g; a source of polyunsaturated fatty acids, PUFA). Heterothermic animals need PUFA to optimize cell function at low *T*_b_ when they enter torpor (Geiser and Heldmaier 1995) and it was found that such supplementation did not affect BMR or capacity for NST of winter-acclimated Siberian hamsters (Gutowski et al. 2011). Hamsters were weighed once a week to ±0.1 g with an electronic balance (SPU402, Ohaus, Parsippany, NJ, USA).

**Fig. 1:**
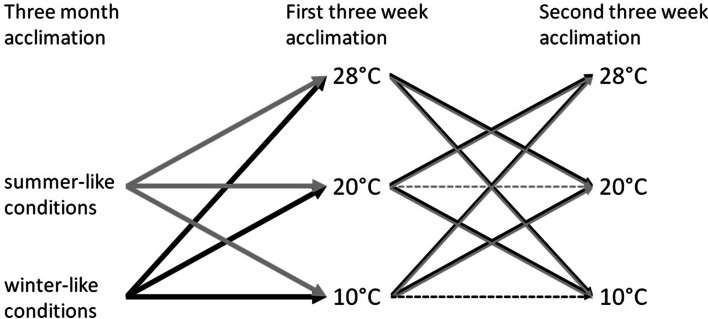
Outline of the acclimation procedure used during winter and summer experiments. The *lines* indicate how animals were moved between ambient temperatures during winter (*black lines*) and summer (*gray lines*) experiments. *Dashed lines* represent individuals kept as a control groups during summer (*gray*) and winter (*black*) experiments. See text for the detailed description

To induce seasonal changes, we acclimated hamsters first to winter- and then to summer-like conditions. At the beginning of the experiment in September 2012 hamsters were acclimated for 3 months to winter-like conditions (short, 8 h photoperiod, lights on at 08:30, *T*_a_ = 10 °C; henceforth, winter conditions or winter experiments, Fig. 1). To test for phenotypic flexibility within seasons we moved hamsters between three acclimation *T*_a_s. After the 3-month acclimation (henceforth, initial acclimation) individuals were randomly assigned to three experimental groups (between 11 and 13 individuals in each group) and moved for ~3 weeks to walk-in climate chambers with *T*_a_s set at 10 ± 2, 20 ± 2 or 28 ± 2 °C, and winter photoperiod (Fig. 1). Similar duration of short-term acclimation was shown to be sufficient to produce phenotypic adjustments of energetics in several mammalian as well as avian taxa (e.g., Li et al. 2001; Nespolo et al. 2002; Rezende et al. 2004; McKechnie et al. 2007; van de Ven et al. 2013). After that, hamsters were again randomly divided, so that six out of 11 individuals kept at 10 °C were moved to *T*_a_ = 20 °C and five to *T*_a_ = 28 °C. Seven out of 12 hamsters acclimated to *T*_a_ = 20 °C were moved to 10 °C and five were moved to 28 °C. Six out of 12 animals kept at *T*_a_ = 28 °C were moved to 10 °C and six were moved to *T*_a_ = 20 °C. Again, each group of animals was acclimated for 3 weeks. After winter experiments, in March 2013 all hamsters were transferred to summer-like conditions for 3 months (long, 16 h photoperiod, lights on at 04:30, *T*_a_ = 20 °C; henceforth, summer conditions or summer experiments). After the 3-month initial acclimation to summer, individuals were randomly assigned to three groups (from 11 to 13 individuals) and moved for ~3 weeks to walk-in climate chambers with *T*_a_s set at 10 ± 2, 20 ± 2 or 28 ± 2 °C and summer photoperiod (Fig. 1). After the first 3-week acclimation to 10, 20 or 28 °C seven individuals out of 13 kept at 10 °C were moved to 20 °C and 6 were transferred to 28 °C. Five out of 11 hamsters acclimated to 20 °C were moved to 10 °C, and 6 to 28 °C. Out of 12 animals kept at *T*_a_ = 28 °C five were moved to 10 °C and 7 to 20 °C. Control groups (*N* = 5 or *N* = 4 randomly selected individuals in winter and summer, respectively) remained under the same, initial conditions, namely *T*_a_ = 10 °C and 8 h photoperiod in winter, and *T*_a_ = 20 °C and 16 h photoperiod in summer. After each 3-month acclimation to seasonal conditions, and within each season, after 3-week acclimations to different *T*_a_s, we measured hamster MR and NST capacity in both experimental and control animals.

### Data collection

Metabolic rate was measured by indirect calorimetry as the rate of oxygen consumption ($\dot V{\text{O}}_ 2$) in an open-flow respirometry systems (Sable Systems International, Las Vegas NV, USA; henceforth: SSI) and all recordings were done in ExpeData (v. 1.43, SSI). Measurements were done during daylight hours between 09:00 and 16:00 CET. During measurements of MR below and within the thermoneutral zone (TNZ) we used two parallel respirometry systems, which allowed us to simultaneously measure MR of 14 individuals. The system was set up so that we measured $\dot V{\text{O}}_ 2$ and the rate of CO_2_ production $(\dot V{\text{CO}}_ 2)$ with FoxBox-C (SSI) in seven individuals, and $\dot V{\text{O}}_ 2$ with FC10a analyzer (SSI) in the remaining seven animals. During all measurements, animals were sealed in 1-L respirometry chambers constructed of translucent polypropylene food containers (HPL 812, Lock&Lock, Hana Cobi, South Korea) covered with paper adhesive tape, and were placed in a custom-modified temperature-controlled cabinet (INCUDIGIT, Selecta, Spain). Temperature in respirometry chambers was measured with type-T thermocouples connected to two eight-channel USB-readers (USB 4718, Advantech Europe, Germany) and was recorded with WaveScan (v. 2.0, Advantech Europe, Germany) on two PC computers. Outside air was compressed using a compressor pump and then it was dried and scrubbed of CO_2_ with a PureGas Generator (Puregas, Westminster, CO, USA). After that, air pressure was reduced to the value slightly exceeding (by ~100 kPa) atmospheric pressure. Flow rate through respirometry chambers was regulated with precise needle valves upstream of the respirometry chambers. Air stream from each chamber was sequentially selected with a multiplexer (MUX SSI) and then flow rate was measured downstream with a mass flow meter (FlowBar-4, SSI). Thereafter the main flow from animals was subsampled at ~100 mL min^−1^, and water vapor pressure of the subsampled air was measured with a water vapor analyzer (RH-300; SSI). Then air was dried with magnesium perchlorate (product number 11636.36, VWR International, Gdańsk, Poland), and subsequently fractional concentrations of the excurrent CO_2_ (FeCO_2_) and O_2_ (FeO_2_) were measured every 2 s using a FoxBox-C integrated O_2_ and CO_2_ analyzer, or only FeO_2_ with a FC-10a O_2_ analyzer (SSI). Gases leaving the respirometry chambers were analyzed for 5 min in each individual, and every 15–20 min (depending on a setup) we did a reference air reading between animals. Depending on a setup this resulted in each animal being measured every 39–44 min. To determine the lower critical temperature (*T*_LC_) and the TNZ in summer- and winter-acclimated hamsters, we measured MR at *T*_a_s between ~6 and ~33 °C. On a particular day, each individual was measured at two, randomly selected *T*_a_s (~3 h at each *T*_a_); these measurements lasted for ~2 weeks in each season. Metabolic rate was calculated from the lowest, stable 2 min of a single $\dot V{\text{O}}_ 2$ recording. Basal metabolic rate was determined as minimum $\dot V{\text{O}}_ 2$ recorded at TNZ at least 5 h after last possible meal, i.e., in post-absorptive phase (Gutowski et al. 2011). When both O_2_ and CO_2_ concentrations were measured, $\dot V{\text{O}}_ 2$ and $\dot V{\text{CO}}_ 2$ were calculated, using equations 11.7 and 11.8 following Lighton (2008). When only O_2_ concentration was measured, $\dot V{\text{O}}_ 2$ was calculated using equation 11.2 (Lighton 2008). Prior to calculating the rate of gas exchange, excurrent air flow rates were corrected for water vapor content using equation 8.6 from Lighton (2008).

Non-shivering thermogenesis was induced by injection of noradrenaline (NA) and gauged as a maximum $\dot V{\text{O}}_ 2$ observed between 5 and 30 min after injection. Hamsters were injected with NA at a dosage of 2.53 *m*_b_ (g)^−0.4^ (mg kg^-1^) (Wunder and Gettinger 1996). In the interest of saving time, we refrained from measuring the metabolic responses of hamsters to a control injection of 0.9 % NaCl solution, a procedure which is typically done to determine whether NA injection results in increased heat production (e.g., Nicol et al. 1997; Golozoubova et al. 2006; Mzilikazi and Lovegrove 2006; Gutowski et al. 2011; Jefimow and Wojciechowski 2014; Stawski et al. 2015). The reason is that, in our previous study (Gutowski et al. 2011), we found that in Siberian hamsters from the same population this dose resulted in NA-induced thermogenesis which was qualitatively and quantitatively different from the response to a control injection of saline solution. Moreover, we carefully inspected each recording to make sure that all hamsters developed a typical thermogenic response to the injection of NA. Maximum $\dot V{\text{O}}_ 2$ after NA injection (Levonor, Polfa-Warsaw, Poland) was measured simultaneously in three hamsters using three parallel open-flow respirometry systems. In one system we measured $\dot V{\text{O}}_ 2$ and $\dot V{\text{CO}}_ 2$, while in the other two we measured only $\dot V{\text{O}}_ 2$. The incurrent flow rate was regulated with a precise needle valve and measured by the mass flow meter (FlowBar-4, SSI) upstream of the respirometry chamber. The air stream was switched between animal chambers and a reference airstream using MUX (SSI) controlled by ExpeData or using the built-in MUX-programming option. Air readings were sampled at 0.5 Hz rate, with 2 min of reference gas readings every 40 min. Before the injection, animals were kept in respirometry chambers at *T*_a_ = 26 °C (~1 °C below *T*_LC_) for 40 min and then after NA injection animals were measured for the next 40 min. Maximum NST was defined as a maximum $\dot V{\text{O}}_ 2$ over a 2-min period after injection of NA. When both concentrations of O_2_ and CO_2_ were measured then $\dot V{\text{O}}_ 2$ and $\dot V{\text{CO}}_ 2$ were calculated using equations 10.6 and 10.7, respectively (Lighton 2008). When we recorded only O_2_, $\dot V{\text{O}}_ 2$ was calculated using equation 10.2 (Lighton 2008).

Hamster *T*_b_ was measured with implantable miniature, thermosensitive data loggers (miniaturized iButtons, models 1921H and 1922L, Dallas Semiconductors, TX, USA) and with thermosensitive, pre-calibrated RFID transponders (Bio-Thermo, Destron Fearing, USA). Loggers measured *T*_b_ continuously whereas RFID transponders were read with a handheld reader (Pocket reader, Destron Fearing, USA) after respirometry measurements at and below TNZ, while animals were still in respirometry chambers. These data were used to calculate minimum *C*. In summer and winter, 3 weeks before metabolic measurements, between 30 and 32 individuals out of 40 were implanted intraperitoneally with miniature thermosensitive data loggers and with RFID transponders. The number of implanted loggers depended on the number of available, fully functional units. Because some of the loggers failed during experiments, we could retrieve a full data set only from 25 individuals, which were measured repeatedly throughout all acclimations in winter and summer (for initial acclimations we retrieved data from 30 individuals). Before implantation, loggers were embedded in paraffin wax, and their final mass ranged between 1.0 and 1.6 g. Animals were implanted under ketamine (40 mg kg^–1^; Narkamon 5 %, SPOFA, Prague, Czech Republic) and xylazine (8 mg kg^–1^; Sedazin 2 %, Biowet, Puławy, Poland) anesthesia. After surgery hamsters recovered for 3 days at *T*_a_ = 20 ± 2 °C. Body temperature was recorded every 20 min with resolution less than 0.2 °C. Logger capacity ranged between 2048 and 4096 samples (depending on the model) and, therefore, they had to be replaced approximately every 55 days. Before all implantations and re-implantations, loggers were calibrated against a traceable mercury-in-glass thermometer in a temperature-controlled ethylene glycol bath (FBC 635, Fisherbrand, Germany).

### Statistical analyses

Using the respiratory exchange ratio (RER =$\dot V{\text{CO}}_ 2/\dot V{\text{O}}_ 2$) obtained from our data or assuming RER = 0.8 (Koteja 1996; using this RER when $\dot V{\text{CO}}_ 2$ is unknown results in smallest calculation error) we calculated metabolic rate in watts (W) using oxyjoule equivalent after Lighton et al. (1987) as follows:

$${\text{MR (W)}} = \frac{{\dot V{\text{O}}_2 (16 + 5.164 \cdot {\text{RER)}}}}{ 6 0},$$

where $\dot V{\text{O}}_ 2$ is oxygen consumption (ml O_2_ min^−1^). Facultative NST (W) was calculated for each individual as a difference between 2-min maximum MR after NA injection and its BMR. Minimum *C* was calculated following Dawson and Schmidt-Nielsen (1966) as:

$$C \, ({\text{W }}^\circ {\text{C}}^{ - 1} {\text{ cm}}^{ - 2} ) { } = \, \frac{\text{MR - EHL}}{{ (T_{\text{b}} { - }T_{\text{a}} )\cdot A_{\text{s}} }},$$

where MR is metabolic rate (W), EHL is evaporative heat loss (W; calculated assuming that evaporation of 1 g H_2_O requires 2.49 kJ), *T*_b_ is body temperature (°C), *T*_a_ is ambient temperature (°C), *A*_s_ is body surface area calculated following Dawson and Hulbert (1970) as *A*_s_ (cm^2^) = 10 *m*_b_^0.67^. Intra-individual variability of *T*_b_ was determined as heterothermy index (HI) following Boyles et al. (2011):

$${\text{HI }}(^\circ {\text{C) }} = \, \sqrt {\frac{{\sum { (T_{b - \bmod } } - T_{b - i} )^2 }}{n - 1}} ,$$

where *T*_b-mod_ is a modal *T*_b_ (°C) of individuals recorded during α-phase, *T*_b-i_ is a *T*_b_ measurement at given time and *n* is the total number of *T*_b_ recordings.

Lower critical temperature was calculated for the relationship between hamster MR and *T*_a_. We used SegReg software (http://www.waterlog.info/segreg.htm; Oosterbaan et al. 1990) to calculate segmented (piecewise) linear regression equations and their breakpoint. The selection of a best fitting function describing the relationship and the breakpoint is done by maximizing the coefficient of determination and testing the significance of the model (Oosterbaan et al. 1990). We report the *T*_LC_ as a breakpoint of the two regression lines ±SE.

### Seasonal changes in response to initial acclimations

Body masses measured after initial acclimation of winter- and summer-acclimated hamsters were compared using paired Student’s *t* test. BMR and fNST of winter- and summer-acclimated hamsters after initial acclimations were compared using repeated measures analysis in a linear mixed effects model (LME) with *m*_b_ as a time-dependent covariate, and season as a fixed factor (IBM SPSS Statistics 21 Command Syntax Reference, p. 1257).

To determine whether BMR and fNST followed the patterns predicted by *m*_b_, both in winter and in summer, we calculated BMR and fNST based on hamster *m*_b_ and compared the differences between predicted and observed values in both seasons. To predict BMR we used regression coefficients published by Lovegrove (2000; “All rodent species: Palearctic” in Table 5): log_10_BMR (mlO_2_ h^−1^) = 1.021 + 0.519 log_10_*m*_b_ (g), and converted the result to W (see above for details of the conversion). Then we calculated deviation from allometrically predicted values of BMR as differences between the observed and predicted BMR and compared them between seasons using paired Student’s *t* test. To predict fNST we first used published regression equations to calculate expected maximum NST for a given *m*_b_ of winter- and summer-acclimated hamsters. Two equations were used; for rodents acclimated to 5 °C: NST (mlO_2_ g^−1^ h^−1^) = 44.7 m_b_ (g)^−0.51^ and to 23 °C: NST (mlO_2_ g^−1^ h^-1^) = 28.9 m_b_ (g)^−0.49^ (Table 3 in Wunder and Gettinger 1996). Again, these results were converted to SI units. Predicted fNST (heat production after NA injection exceeding BMR) was calculated by subtracting allometrically predicted BMR (see above) from the expected maximum NST calculated after Wunder and Gettinger (1996). Then we calculated differences between observed and predicted fNST and compared these deviations between summer- and winter-acclimated hamsters using paired Student’s *t* test. The same test was used to compare minimum *C* between winter and summer acclimations. Modal *T*_b_s of winter- and summer-acclimated hamsters were compared with two-sided Wilcoxon paired test because of the relatively small sample size (*N* = 30; individuals measured repeatedly in winter and in summer) and the lack of normal distribution, which could not be achieved by any transformation. Box–Cox-transformed HIs calculated from *T*_b_s collected during initial acclimations were compared between summer and winter using paired Student’s *t* test. Additionally we analyzed HIs calculated for 25 individuals for which we had repeated measurements for the whole study. These HIs were Box–Cox transformed prior to the analysis and were compared in general linear model (GLM) between winter and summer, with acclimation as a fixed factor, the interaction between season and acclimation as another independent variable, and *T*_a_ as a covariate. To account for repeated measurements of each hamster, the animal ID was included as a random factor.

### Phenotypic flexibility within seasons

We estimated phenotypic flexibility of *m*_b_, BMR, fNST and *C* as a difference between data collected after first and second 3-week acclimation treatments to 10, 20 or 28 °C in each season. These differences were analyzed against a change in *T*_a_ between these two acclimations. Seasonal differences in intra-individual variation of *m*_b_ between acclimation treatments were analyzed in LME with *m*_b_ as time-dependent covariate, change of *T*_a_ as a covariate, season as a fixed factor and interaction between season and change of *T*_a_. We used repeated measures analysis in LME, with change in *m*_b_ and the absolute *m*_b_ of summer- and winter-acclimated hamsters as time-dependent covariates, change in *T*_a_ as a covariate, season as a fixed factor and the interaction between change in *T*_a_ and season as another independent variable, to examine intra-seasonal changes in BMR, fNST and *C* in response to acclimation to various *T*_a_s between summer and winter. To determine how BMR and fNST changed seasonally with regard to the direction of changes in acclimation *T*_a_s we also compared the percent change in BMR and fNST per 1 °C. We did so for both, summer and winter. The percent change in BMR and fNST in response to changes in *T*_a_ was calculated in relation to the values measured after initial acclimation. The calculated values were then related to change in *T*_a_, which occurred between particular acclimation treatments and was expressed as a percent per 1 °C. The results from summer and winter were compared using Mann–Whitney *U* test separately for the increase or for the decrease of *T*_a_.

### Acclimation history and reversibility of changes

To account for the possible effect of the duration of acclimation, two different control groups were kept continuously under winter- or summer-like conditions. In each season different individuals were randomly selected to control groups. In winter the control group consisted of five, and in summer of four individuals. Because BMR and fNST of these individuals were not normally distributed and sample sizes were small, we compared BMR, fNST, *C* and *m*_b_ between three consecutive periods (initial, first short and second short acclimations) using Friedman repeated measures test with Wilcoxon pairwise test for post hoc comparisons.

We examined changes in BMR, fNST, *C* and *m*_b_ to test whether changes in the phenotype were reversible (*sensu* Piersma and Drent 2003). These traits were compared in hamsters that were acclimated to the same *T*_a_ during initial and second short-term acclimation treatments (10 °C in winter, *N* = 12, or 20 °C in summer, *N* = 14). We used two-way RM-ANOVA with change of *T*_a_ between measurements as a between-subject factor (during winter acclimation: increase by 10 or 18 °C and during summer decrease by 10 °C or increase by 8 °C). Prior to the analysis *m*_b_ was Box–Cox transformed.

All data were analyzed using SPSS v.21 (IBM Corp. 2012). With the exception of data for seasonal differences in fNST, all data in the text were presented as mean ± SD. Seasonal differences in fNST were presented as estimated marginal means from the LME ± SE. The degree of phenotypic changes in BMR and fNST were presented as regression coefficients ± SE of the relationship between change in BMR or fNST and the change in acclimation *T*_a_ as an independent variable. Significance was accepted at *P* ≤ 0.05.

## Results

### Seasonal changes in response to initial acclimations

**Fig. 2:**
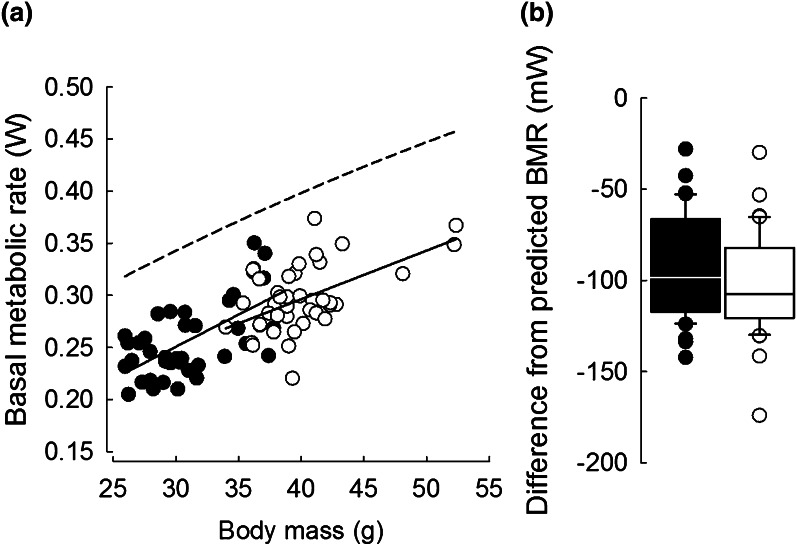
**a** Relationships between basal metabolic rate and body mass in hamsters acclimated to winter- (*black*) and summer-like (*white*) conditions. *Dashed line* indicates basal metabolic rate predicted by body mass following Lovegrove (2000). **b** Deviation from allometrically predicted values of BMR in hamsters acclimated to winter- (*black*) and summer-like (*white*) conditions. *Line* median, *box* 25–75 %, *whiskers* 10–90 %. Note different scales on each plot

Overall, *m*_b_ of hamsters after initial acclimation to winter (31.6 ± 3.82 g) was ~22 % lower than after initial acclimation to summer (40.6 ± 3.81 g; *t* = 12.55, *P* < 0.001, *N* = 40; see Table 1 and Table 1a in App. 1 for mean *m*_b_ of hamsters in each acclimation group). The *T*_LC_ for hamsters acclimated to winter was ~1.7 °C lower (26.9 ± 0.3 °C) than of hamsters acclimated to summer-like conditions (28.6 ± 0.2 °C; Fig. S1 in App. 1). Whole animal BMR was ~13 % lower after acclimation to winter (0.26 ± 0.04 W) than after acclimation to summer (0.30 ± 0.03 W; Table 1 and Table 2a in App. 1). However, it correlated positively with hamster *m*_b_ (*F*_1,74.6_ = 44.20, *P* < 0.001; Fig. 2a) and after taking that into account, BMR did not differ between winter and summer (*F*_1,65.1_ = 0.96, *P* = 0.33; Fig. 2a). BMR was lower than expected from the allometric relationship between BMR and *m*_b_ for rodents (Lovegrove 2000) both for winter (*t* = 20.04, *P* < 0.001, *N* = 40) and for summer-acclimated hamsters (*t* = 23.64, *P* < 0.001, *N* = 40). However, the difference between predicted and expected BMR was 10 % greater after acclimation to summer (−101.35 ± 26.78 mW) than after acclimation to winter (−91.16 ± 28.41 mW; *t* = 2.23, *P* = 0.032, *N* = 40, Fig. 2a and b).

**Fig. 3:**
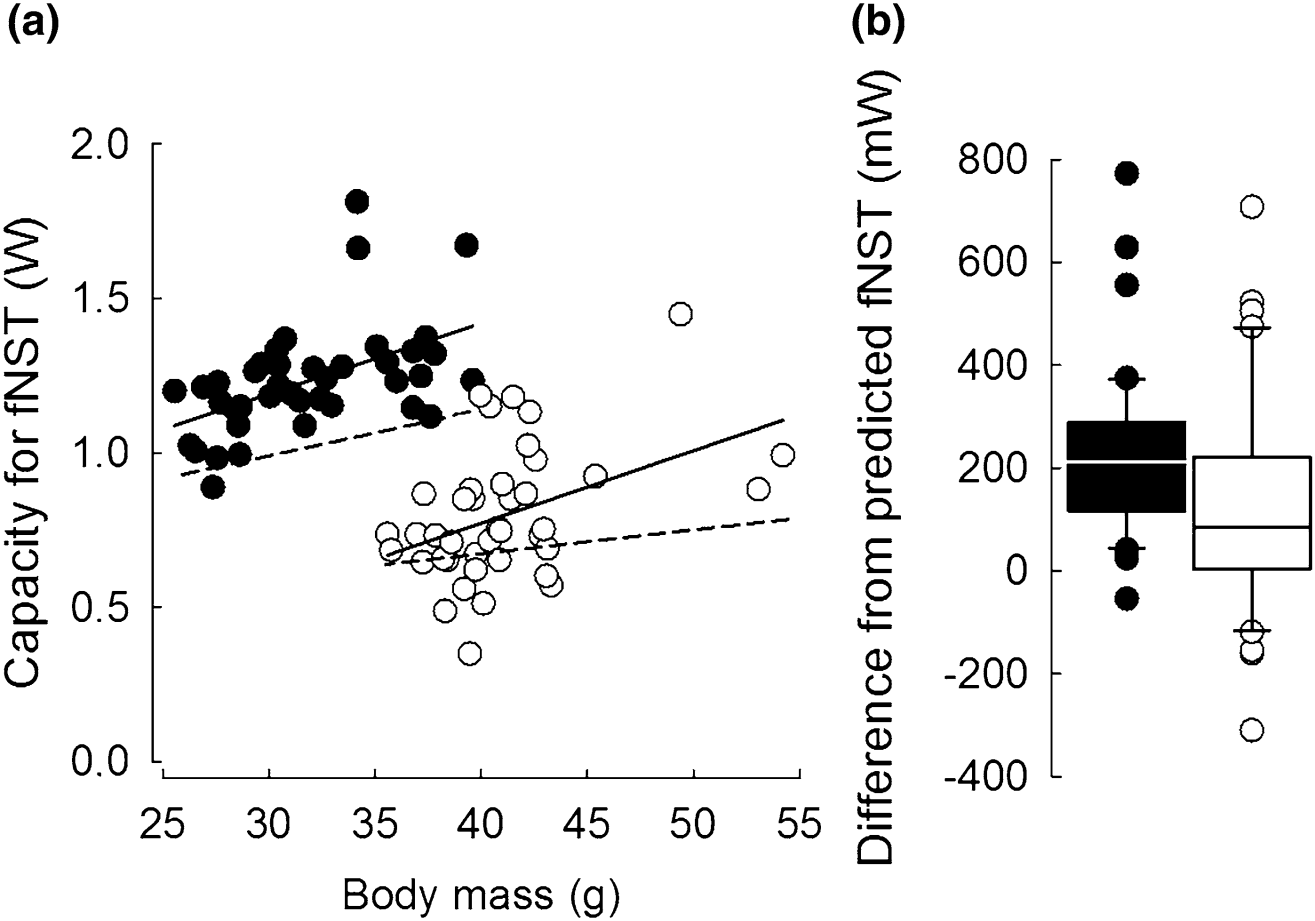
**a** Relationships between capacity for facultative non-shivering thermogenesis (fNST) and body mass in hamsters acclimated to winter- (*black*) and summer-like (*white*) conditions. *Dashed lines* indicate fNST predicted by body mass and acclimation temperature following Wunder and Gettinger (1996; for details see “Seasonal changes in response to initial acclimations” section). **b** Deviation from allometrically predicted values of fNST in hamsters acclimated to winter- (*black*) and summer-like (*white*) conditions. *Line* median, *box* 25–75 %, *whiskers* 10–90 %. Note different scales on each plot

Capacity for fNST correlated positively with *m*_b_ (*F*_1, 67.7_ = 21.79, *P* < 0.001, Fig. 3a; Table 1 and Table 3a in App. 1). After adjusting for *m*_b_, fNST was markedly higher after winter than after summer acclimation (*F*_1, 72.6_ = 110.74, *P* < 0.001; Fig. 3a). At *m*_b_ = 36.51 g, which was the central *m*_b_ for the analyzed data set, capacity for fNST after winter acclimation (1.34 ± 0.04 W) was ~49 % higher than fNST capacity after summer acclimation (0.69 ± 0.04 W). Facultative NST of winter-acclimated hamsters was higher than allometrically expected for animals acclimated to 5 °C (Wunder and Gettinger 1996; *t* = 9.01, *P* < 0.001, *N* = 40). Also, in summer-acclimated hamsters fNST was higher than fNST expected for animals acclimated to 23 °C (Wunder and Gettinger 1996; *t* = 3.81, *P* < 0.001). Likewise, the difference between fNST measured in the present study and expected from *m*_b_ and acclimation temperature (Wunder and Gettinger 1996) was higher after acclimation to winter (225.26 ± 156.21 mW) than to summer (124.37 ± 204.00 mW; *t* = 2.61, *P* = 0.013; Fig. 3b).

**Fig. 4:**
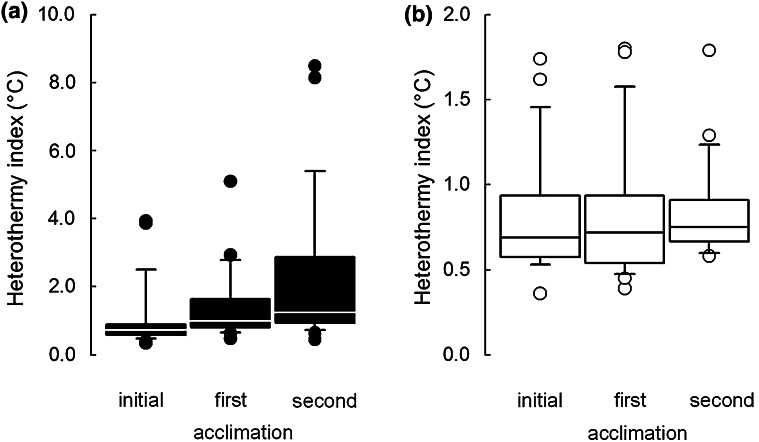
Heterothermy indices in hamsters acclimated to winter- (**a**, *black*) and summer-like (**b**, *white*) conditions. Heterothermy indices were calculated for body temperatures of hamsters collected during 3-month initial acclimation to winter- or summer-like conditions and during subsequent first and second 3-week acclimations within each season. See text for more details. *Line* median, *box* 25–75 %, *whiskers* 10–90 %. Note different scales on each plot

There was no difference in *C* of hamsters between winter (0.25 ± 0.07 mW °C^−1^cm^−2^) and summer acclimations (0.25 ± 0.04 mW °C^−1^cm^−2^; *t* = 0.50, *P* = 0.62, *N* = 40; Table 1 and Table 4a in App. 1). Modal *T*_b_ during the α-phase after initial winter acclimation (35.97 ± 0.30 °C) was ~0.6 °C lower than after summer acclimation (36.62 ± 0.57 °C; *Z* = 4.27, *P* < 0.001, *N* = 30). After initial acclimations to summer or winter HI did not differ between seasons (*t* = 0.61, *P* = 0.55, *N* = 30). Nevertheless, when all data from summer and winter were pooled, HI of 25 hamsters measured repeatedly over the course of experiment was not related to acclimation *T*_a_ (*F*_1,119_ = 0.78, *P* = 0.38) but was different between individuals (*F*_24,119_ = 2.35, *P* = 0.001), and was higher in winter than in summer (*F*_1,119_ = 25.15, *P* < 0.001). Namely, heterothermy developed during winter experiments (*F*_2,119_ = 9,16, *P* < 0.001 for the interaction between season and acclimation) and was highest after the second short acclimation (Fig. 4a). In total 56 % of 25 individuals measured repeatedly throughout the study entered torpor at least once, and the lowest *T*_b_ of winter-acclimated hamsters observed in this study was 16.6 °C in hamsters acclimated to *T*_a_ = 10, and 23.3 °C in those acclimated to 20 °C. In hamsters acclimated to 28 °C the lowest recorded *T*_b_ in winter was 31.9 °C. In contrast, variability of *T*_b_ in summer was relatively stable throughout the season (Fig. 4b).

### Phenotypic flexibility within seasons

During winter experiments hamster *m*_b_ decreased continuously with the progress of the acclimations and was ~9 % lower after the second short-term acclimation than after initial acclimation (~6 weeks apart; Table 1 and Table 1a in App. 1). In contrast, in summer *m*_b_ of individuals did not differ between consecutive acclimations (Table 1 and Table 1a in App. 1). Individual variations in hamster *m*_b_ during short-term acclimations were not related to changes in acclimation *T*_a_s (*F*_1,69.7_ = 0.94, *P* = 0.34) or to an average *m*_b_ of individuals (*F*_1,60.8_ = 1.62, *P* = 0.21), and season did not change it (no interaction between season and change of *T*_a_; *F*_1,74.6_ = 2.51, *P* = 0.12).

**Fig. 5:**
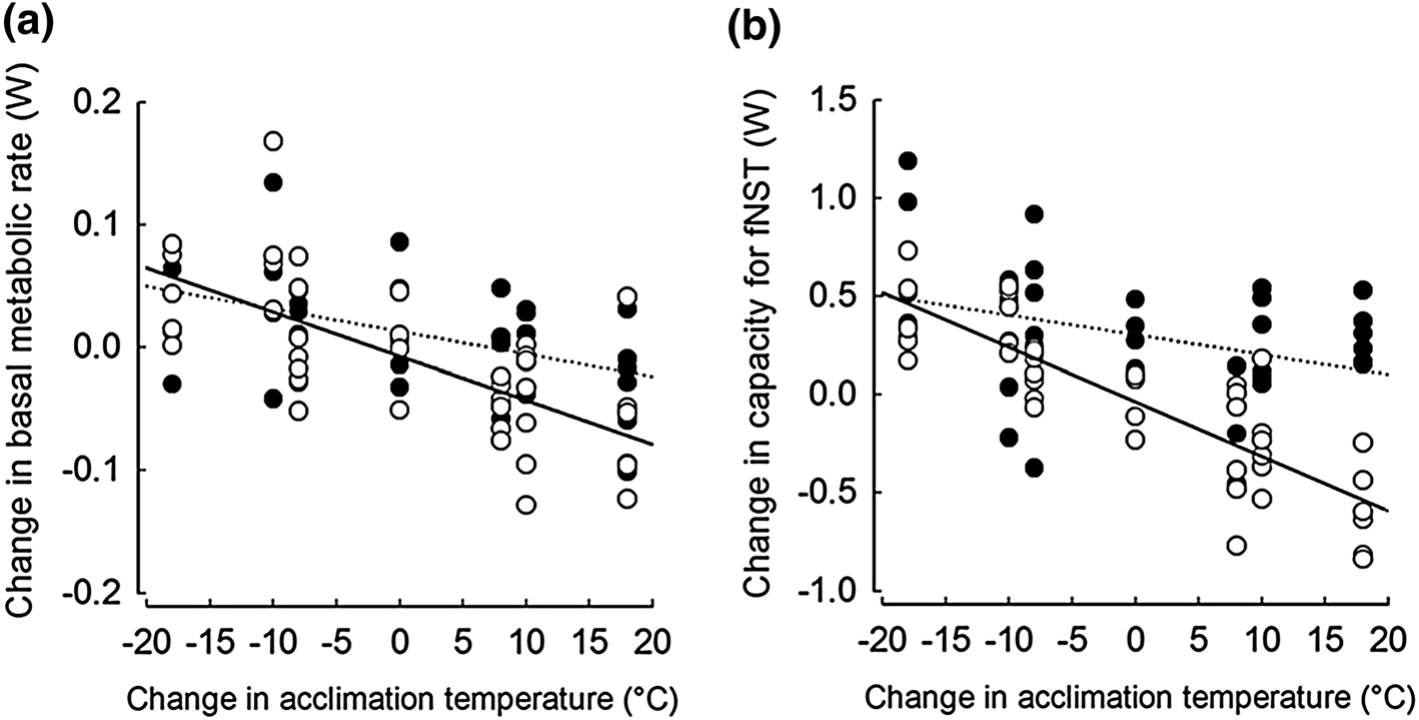
Relationships between change in basal metabolic rate (**a**), change in capacity for facultative non-shivering thermogenesis (fNST; **b**) and change in acclimation temperatures in winter (*black points*, *dotted line*)- and summer (*white points*, *solid line*)-acclimated hamsters

Changes of BMR after short-term acclimations did not correlate neither with *m*_b_ of individuals (*F*_1,61.8_ = 0.06, *P* = 0.82) nor with intra-individual variations in *m*_b_ (*F*_1,73.9_ = 0.02, *P* = 0.88). They were, however, negatively related to changes in acclimation *T*_a_ (*F*_1,72.0_ = 41.88, *P* < 0.001) and this relationship differed between seasons (significant interaction between acclimation *T*_a_ and season; *F*_1,74.0_ = 4.98, *P* = 0.029, Fig. 5a). As a result, flexibility of BMR was approximately two times higher in summer-acclimated hamsters (−3.53 ± 0.64 mW change per 1 °C change in *T*_a_) when compared to winter-acclimated animals (−1.84 ± 0.54 mW change per 1 °C change in *T*_a_; Fig. 5a). Moreover, there was no difference between summer (0.9 ± 1.8 %)- and winter (1.2 ± 1.7 %)-acclimated hamsters in percent change of BMR per 1 °C decrease in *T*_a_ (*U* = 115, *z* = −0.47, *P* = 0.64). There was, however, a significant difference between summer- and winter-acclimated hamsters when *T*_a_ increased between acclimations (*U* = 64, *z* = −2.76, *P* = 0.006). Summer-acclimated hamsters changed their BMR by −1.5 ± 1.2 % °C^−1^ while winter-acclimated hamsters did not (average change: −0.1 ± 1.3 % °C^−1^).

Change in fNST in response to change in acclimation *T*_a_s was not related to *m*_b_ of individuals (*F*_1,67.0_ = 0.13, *P* = 0.72) nor to changes in *m*_b_ (*F*_1,72.5_ = 1.68, *P* = 0.20). However, both in summer and winter, changes in fNST were negatively related to change in acclimation *T*_a_ (*F*_1,73.9_ = 65.36, *P* < 0.001) and this relationship differed seasonally (*F*_1,72.1_ = 13.10, *P* = 0.001) being ~65 % greater in summer-acclimated hamsters (−27.62 ± 3.076 mW change per 1 °C change in *T*_a_) than in winter-acclimated individuals (−9.27 ± 4.14 mW change per 1 °C change in *T*_a_, Fig. 5b). There was no difference between summer (3.1 ± 2.5 % °C^−1^)- and winter (2.9 ± 3.2 % °C^−1^)-acclimated hamsters in the percent change in fNST per 1 °C change when *T*_a_ decreased (*U* = 126, *z* = −0.06, *P* = 0.96). It did, however, differ seasonally when *T*_a_ increased (*U* = 30, *z* = −3.93, *P* < 0.001). The change in fNST per 1 °C change in *T*_a_ was smaller in winter (1.1 ± 2.1 % °C^−1^) than in summer (−3.9 ± 2.9 % °C^−1^).

Within each season there was no relationship between intra-individual variations in *C* and *m*_b_ of an individual (*F*_1,56.6_ = 0.30, *P* = 0.60) as well as between variations in *C* and intra-individual variations in *m*_b_ (*F*_1,57.7_ = 0.37, *P* = 0.55). Variations in *C* were also not related to change in *T*_a_ between acclimations (*F*_1,59.8_ = 1.16, *P* = 0.29) and it did not differ seasonally (*F*_1,54.3_ = 0.27, *P* = 0.61).

### Acclimation history and reversibility of changes

Individuals from a control group, which were kept continuously at 10 °C in winter consistently decreased *m*_b_ from initial to second short-term acclimation (Table 1). Hamsters kept under constant thermal conditions in summer (*T*_a_ = 20 °C) maintained stable *m*_b_ (Table 1). Although fNST capacity also changed in control animals in winter, there was no consistent trend in these changes from an initial to second acclimation (Table 1). Hamsters marginally decreased fNST from initial to first acclimation (*P* = 0.09) and increased it from first to second short acclimation (*P* = 0.09). Facultative NST was, however, stable in individuals from control group in summer (Table 1). Hamsters from control groups did not change their BMR or *C* neither in winter nor in summer (Table 1).

**Table 1:** Body mass (*m*_b_), basal metabolic rate (BMR), facultative non-shivering thermogenesis (fNST) and minimum thermal conductance (*C*) of Siberian hamsters, *Phodopus sungorus* randomly assigned to control groups (*N* = 5 or *N* = 4 in winter and summer, respectively) which were maintained in constant *T*_a_ during winter (10 °C) and summer (20 °C) experiments

Season	Trait	Acclimation			*χ* ^2^	*P*
		Initial	First	Second		
Winter	*m*_b_ (g)	31.71 ± 4.51	29.67 ± 4.89	27.87 ± 4.68	9.42	<0.001*
	BMR (W)	0.25 ± 0.04	0.22 ± 0.03	0.24 ± 0.03	2.50	0.367
	fNST (W)	1.38 ± 0.33	1.05 ± 0.23	1.32 ± 0.19	7.12	0.024*
	*C* (mW °C^−1^ cm^−2^)	0.24 ± 0.06	0.28 ± 0.01	0.25 ± 0.02	1.35	0.522
Summer	*m*_b_ (g)	39.48 ± 1.75	40.55 ± 1.89	41.48 ± 2.05	1.65	0.431
	BMR (W)	0.28 ± 0.04	0.26 ± 0.04	0.26 ± 0.05	3.06	0.273
	fNST (W)	0.88 ± 1.00	1.07 ± 0.17	1.03 ± 0.29	1.65	0.431
	*C* (mW °C^−1^ cm^−2^)	0.25 ± 0.05	0.22 ± 0.04	0.21 ± 0.03	1.65	0.431

Values were compared using Friedman repeated measures test with Wilcoxon pairwise test for post hoc comparisons. Values are presented as mean ± SD, χ^2^—value of the test statistics

* Post hoc comparisons showed differences between means at 0.1 > *P* > 0.05

BMR of individuals exposed to the same *T*_a_ during the initial, 3-month acclimation and during the second short-term acclimation, after three intervening weeks at different *T*_a_s (20 or 28 °C in winter, or 10 or 28 °C in summer), did not differ between initial and second short-term acclimation, neither in winter (*F*_1,10_ = 0.01, *P* = 0.92) nor in summer (*F*_1,12_ = 0.95, *P* = 0.349). We also did not find significant interactions between different *T*_a_s and reversibility of BMR during winter (*F*_1,10_ = 0.44, *P* = 0.52) or summer (*F*_1,12_ = 1.70, *P* = 0.22) experiments. The capacity for fNST measured in hamsters from these groups did not differ, neither in winter (*F*_1,10_ = 0.82, *P* = 0.39) nor in summer (*F*_1,12_ = 0.21, *P* = 0.65). There was also no interaction between different *T*_a_s and reversibility of capacity for fNST during winter (*F*_1,10_ = 0.45, *P* = 0.52) and summer experiments (*F*_1,12_ = 2.38, *P* = 0.15). Likewise, *C* was similar before and after ~3-week exposure to different *T*_a_s, both in winter (*F*_1,10_ = 2.10, *P* = 0.18) and in summer (*F*_1,12_ = 0.36, *P* = 0.56). There was no interaction between reversibility of *C* and acclimation to different *T*_a_s during winter (*F*_1,10_ = 0.32, *P* = 0.58) and summer experiments (*F*_1,12_ = 0.70, *P* = 0.42). There was also no interaction between differences in *m*_b_ and changes in *T*_a_ to which animals were acclimated during winter (*F*_1,10_ = 0.69, *P* = 0.43) and summer experiments (*F*_1,12_ = 0.15, *P* = 0.71). However, *m*_b_ of these hamsters differed between these two acclimations, both in winter (*F*_1,10_ = 13.68, *P* = 0.004) and in summer (*F*_1,12_ = 5.37, *P* = 0.039). During winter experiments, *m*_b_ of individuals which in the interim were exposed for ~3 weeks to 20 °C decreased by 8 %, while a 9.5 % decrease of *m*_b_ was observed in individuals acclimated to 28 °C between initial and final acclimations (App. 1). During summer experiments, animals which were exposed to 10 °C increased their *m*_b_ by ~5.5 % while those acclimated to 28 °C only by ~3.0 % (App. 1).

## Discussion

Presented results support our hypothesis that phenotypic flexibility of the main mechanisms of heat production in response to changes in thermal environment differs seasonally and is greater in summer than in winter. We found that both summer- and winter-acclimated Siberian hamsters showed reversible changes in BMR and fNST when exposed to short-term changes in thermal environment despite the fact that *m*_b_ did not change correspondingly. At the same time, minimum *C* did not change between and within seasons. To the best of our knowledge, this is the first report of seasonal variations in phenotypic flexibility of energetics measured in the same individuals across seasons.

Although whole animal BMR differed considerably between seasons, these differences resulted mainly from seasonal changes in *m*_b_ and were not accompanied by changes in *C*. The lack of increase in *C* despite the lower *m*_b_ in winter agrees with results published by Heldmaier and Steinlechner (1981a). Thus, the lack of change in *C* would be a combined effect of a decreased mass of subcutaneous fat (Wade and Bartness 1984), increased fur depth and its density (Heldmaier and Steinlechner 1981a; Paul et al. 2007), lowered *m*_b_, and possibly also of regulating a slightly lower *T*_b_ (Heldmaier 1989 and present results). Nonetheless, present results indicate that short changes of *T*_a_, even its increase by 18 °C, did not affect hamster *C*, and it was true both for summer and winter. We argue that intra-seasonal flexibility of Siberian hamster phenotype in response to variations in thermal environment appears only in mechanisms of heat production, i.e., BMR and fNST.

Seasonal changes of BMR were mainly a consequence of changes in hamster *m*_b_ what is in line with results of other studies on seasonal changes of *m*_b_ and energy metabolism of small mammals (for review see: Heldmaier 1989; Lovegrove 2005). Winter decrease in *m*_b_ and whole animal BMR are considered an adaptation allowing to decrease the total costs of living of small animals in winter when energy resources are limited (Heldmaier and Steinlechner 1981a, Bozinovic et al. 1990). In Siberian hamsters seasonal changes in *m*_b_ were associated mainly with decreasing fat mass (Wade and Bartness 1984), yet in other taxa exposure to short photoperiod or winter acclimatization leads also to decrease in lean mass, including the mass of digestive tract organs (Lynch 1973; Bozinovic et al. 1990; note, however, that Bozinovic et al. found an increase in both mass and length of small intestine in winter-acclimatized *Abrothrix andicus*). Although in some species flexibility of MR was related to, or even resulted from, changes in *m*_b_ during short-term, abrupt changes in *T*_a_ (Williams and Tieleman 2000; van de Ven et al. 2013), regardless of the season *m*_b_ of hamsters did not change in response to intra-seasonal variations in *T*_a_ (short term acclimations). This is especially important since in winter hamster *m*_b_ was decreasing continuously with consecutive acclimations while in summer it was relatively stable (Table 1 and Table 1a in App. 1). Winter decrease in *m*_b_ is a typical response of Siberian hamsters to short photoperiod (Scherbarth and Steinlechner 2010) and it reaches its nadir after ~16–18 weeks (Masuda and Oishi 1995; Elliott et al. 1987). At the same time, at 16–18 weeks of acclimation to winter-like conditions, torpor frequency and the capacity for fNST reach their maximum (Elliott et al. 1987; Jefimow et al. 2004). This was the time when we did our experiments. Thus, in all analyses, we accounted for both intra- and inter-individual variation in *m*_b_ and found that they were not related to changes of hamster energetics in response to short-term acclimations. Hence, it is unlikely that the winter decrease of *m*_b_ would affect results of our analyses of phenotypic flexibility. However, in the present study, the lack of flexible changes in hamster *m*_b_ did not affect flexibility of other traits, like BMR or fNST. Similar results were obtained for several rodent species in which *m*_b_ did not correlate with changes in energetics (Li et al. 2001; Chi and Wang 2011). Body mass is considered the main predictor of the whole animal BMR (White and Seymour 2003). This is because *m*_b_ is a sum of the mass of all tissues including those which build metabolically active organs, e.g., gastrointestinal tract, heart, kidneys (Daan et al. 1990; Williams and Tieleman 2000). Thus, animals may manipulate the mass of different body components to achieve similar *m*_b_. For example, Piersma and Jukema (2002) found similar change in *m*_b_ of golden plovers *Pluvialis apricaria* despite seasonal differences in body composition. Namely, spring birds amassed mainly proteins, whereas in autumn fat dominated, yet, total *m*_b_ of birds in spring and in autumn was nearly equal (Piersma and Jukema, 2002). It was also found that greater changes in *m*_b_ of dark-eyed juncos *Junco hyemalis* acclimated to long photoperiod (Swanson et al. 2014) did not correlate with increase of their summit MR (*M*_sum_; Stager et al. 2015). Stager et al. (2015) at the same time found that similar increase in *M*_sum_ after cold acclimation was realized via different transcriptomic avenues in short- and long-day exposed birds. The above avian examples could indicate that changes in BMR and fNST of hamsters which were not paralleled by changes in *m*_b_ could have resulted from a different orchestration of the animal energy balance, possibly by changes in the mass of various organs or of different expression of genes coding enzymes involved in the maintenance of energy balance (Książek et al. 2009; Stager et al. 2015). This suggests that changes at each level of phenotypic organization may affect the scope for the flexibility of energetics and do not necessarily have to be reflected in the change of *m*_b_ as a whole.

We found that Siberian hamsters maintained lower BMR than allometrically predicted based on equations for small Palearctic rodents (Lovegrove 2000). This difference was greater in hamsters acclimated to summer than to winter (Fig. 2a and b) what agrees with results of Heldmaier and Steinlechner (1981a) who observed higher mass-specific BMR in winter-acclimated individuals. This seasonal difference correlates with seasonal differences in their phenotypic flexibility. It indicates that, for a given *m*_b_, winter-acclimated animals regulated their energy expenditure at a higher level than summer-acclimated ones, probably by smaller reduction of metabolically active tissues compared to the decrease of fat mass (Klingenspor et al. 2000). The lower *T*_a_ and the less flexible energetics of hamsters in winter suggest that thermal conditions during seasonal acclimation could have affected subsequent reaction norm for changes in BMR. It is possible that, relative to their *m*_b_, hamsters acclimated to 10 °C in winter had higher MR and higher mass of metabolically active organs than hamsters acclimated to summer-like conditions. Although photoperiod is the primary factor influencing the seasonal acclimation in Siberian hamsters (Heldmaier and Steinlechner 1981a, b), the lower acclimation *T*_a_ that we exposed the animals to might have resulted in increased BMR (Wiesinger et al. 1989). This might occur as in Brandt’s voles Lasiopodomys brandti in which an increase in BMR was correlated with increased mass of metabolically active organs (Song and Wang 2006). Also a study by Puchalski et al. (1987) shows that in winter photoperiod, mass of metabolically active organs was slightly, but significantly, higher in Siberian hamsters acclimated to variable cold (−2 to 12 °C) than to 23 °C. Finally, rufous-collared sparrows *Zonotrichia capensis* first acclimated to 15 °C and then moved to 30 °C changed their BMR less than individuals acclimated in the reverse order (Barceló et al. 2009). The same study showed that reduction of the mass of metabolically active organs in response to rise in *T*_a_ was slower than their synthesis in response to decrease in *T*_a_ (Barceló et al. 2009). Similar mechanism could be responsible for lower flexibility of heat production (here, BMR and fNST) in winter-acclimated hamsters. Hamsters acclimated to cold would need more time to adjust their phenotype (or simply change the mass of their organs) in response to short-term acclimation to new conditions than animals previously acclimated to 20 °C. When exposed to decreasing *T*_a_ in winter hamsters increased their BMR as during summer acclimation. There were, however, significant differences in response to short-term acclimations when *T*_a_ increased. In summer, hamsters decreased BMR when *T*_a_s raised, while in winter they were unable to do so. We propose that changes in energetics in response to seasonal acclimatization to moderate *T*_a_s in summer would allow animals to adjust their metabolism much faster and to greater extent than in animals seasonally acclimatized to lower *T*_a_s. Since under natural conditions animals acclimatized to winter are exposed to both short photoperiod and cold, this combination might lead to lower flexibility of rodent BMR. However, maintaining basal heat production on the constant level could result in faster depletion of energy reserves.

The most important and the most efficient mechanism of heat production during acclimation to cold is NST (Janský 1973; Merritt 1986; Merritt et al. 2001). On the one hand, hamsters acclimated to short days and 10 °C had much higher capacity for fNST than expected from *m*_b_ for animals acclimated to 5 °C. On the other hand, hamsters acclimated to long days and 20 °C showed similar capacity for fNST to that expected for animals acclimated to 23 °C (Fig. 3b). This indicates that seasonal differences in the capacity for fNST (Fig. 3a) resulted from both acclimation to cold and short photoperiod. In short days prolonged secretion of melatonin mediates the seasonal increase of NST capacity (Heldmaier et al. 1981, 1982; Heldmaier and Lynch 1986; Bartness et al. 2002). This role of melatonin in the development of NST could explain why fNST was less flexible in winter. Despite short-term changes in *T*_a_, consistently long duration of melatonin secretion might have prevented *T*_a_-associated changes in the capacity for fNST; especially the decrease of fNST as *T*_a_ increased (Fig. 5b). This could offer potential benefits by maintaining the most effective way of heat production in face of the upcoming cold (Janský 1973) as well as the capacity to rewarm from torpor (Jefimow et al. 2004). At the same time, it could lead to added cost of maintaining unnecessarily high capacity of facultative heat production. A similar mechanism might also offer a more general explanation for the lesser winter flexibility of animal energetics. Short photoperiod affects melatonin secretion which binds to the receptors in the suprachiasmatic nuclei of the hypothalamus (Bartness et al. 2002). This structure of the brain controls directly and indirectly many peripheral effectors, among them brown adipose tissue, and possibly also adrenal cortex (Bartness et al. 2001). The latter one is involved in the control of seasonal changes of metabolism (Scherbarth and Steinlechner 2010), and perhaps of its flexibility. An indirect support for this hypothesis would be offered by an attenuated release of glucocorticosteroids (GC) in response to cold stress in deer mice *Peromyscus maniculatus* acclimated to short photoperiod (Demas and Nelson 1996) and lower variability of MR in Gambel’s white-crowned sparrows *Zonotrichia leucophrys gambelii* which were artificially exposed to increased concentration of GC’s (Buttemer et al. 1991).

One could argue though that a lesser flexibility of winter-acclimated Siberian hamsters could result from an addition of sunflower seeds to their diet or from the fact that hamsters differed in age between seasons. In our opinion, both possibilities are rather unlikely. First, Gutowski et al. (2011) found no effect of sunflower seeds on BMR or on the capacity for fNST in Siberian hamsters. Second, although our hamsters were 4 months older (~8–12 months old) during summer than during winter acclimation (~4–6 months old), they were still adult and could not be regarded as “old individuals” with impaired thermoregulation (cf. Gordon 1993). One-year-old hamsters do not differ in thermogenic capabilities from 3- to 4-month-old individuals (Heldmaier and Steinlechner 1981a). Although there are data showing that Siberian hamsters kept constantly at 23 °C and under natural photoperiod did not use torpor in their second winter, while individuals kept outside, under natural photoperiod and *T*_a_, did so (Heldmaier and Steinlechner 1981b), it was suggested that older hamsters require additional environmental signals, like change in *T*_a_, to enter torpor (Heldmaier and Steinlechner 1981b). We are not aware of any literature data on the influence of age on flexibility of animal energetics in response to changes in *T*_a_ or photoperiod, or both. Nevertheless, if age would affect hamster flexibility, e.g., due to impaired thermoregulation or perception of the environmental cues, then we should rather expect the opposite, i.e., smaller flexibility in summer, when hamsters were older.

In the population of Siberian hamsters there is a proportion of individuals which do not respond to seasonal changes in photoperiod (Lynch et al. 1989). In line with that, more than half of hamsters studied developed heterothermy while others did not enter torpor even once. Because both heterothermy and phenotypic flexibility carry costs (DeWitt et al. 1998; Humphries et al. 2003), one might expect a trade-off between the flexibility of energetics and the use of torpor. If so, then heterothermy as a rapid energy-conserving response could serve as a potential alternative for the phenotypic flexibility of energetics. The lower flexibility of BMR and fNST that we observed in winter acclimated hamsters, in fact, partially supports this hypothesis. However, to properly test it directly, one would need to compare phenotypic flexibility of responding and non-responding hamsters exposed to identical variations in thermal environment.

Our results on seasonal variations in phenotypic flexibility correlate also with seasonal differences in daily temperature variability observed in Siberia, the natural habitat of Siberian hamsters. Indeed, data from several locations in Siberia indicate that in the winter months, *T*_a_ is less variable than in summer (WWIS 2015). Thus, our results corroborate the climatic variability hypothesis (Janzen 1967; Stevens 1989; Ghalambor et al. 2006), but on a timescale rather than on a geographical scale. Since more variable physiological functions evolved in more variable climates (Naya et al. 2008, 2012), one could predict that more variable conditions in summer favored more flexible phenotypes, and more fixed ones in winter, when energy conservation in harsh but stable conditions was most beneficial.

### Inference and a perspective

The results of the present study indicate that energetics of small, photoresponsive mammals is less flexible in winter than in summer. We argue that understanding the seasonal changes in phenotypic flexibility and its mechanistic basis is crucial for predicting the biological consequences of global climate change and its potential impact on natural populations of animals. In the present study, Siberian hamsters acclimated to winter-like conditions were unable to lower their obligatory and facultative heat production in response to increasing *T*_a_. Contrary to that, summer-acclimated hamsters adjusted their energetics irrespective of the direction of the *T*_a_ change. Assuming that present results are valid for other endothermic taxa, there are two possible outcomes of this situation. First, not taking into account seasonally different reaction norms for phenotypic flexibility of energetics might result in incorrect predictions for animal responses to changing climate. Second, and more important, consequences of the increased probability of weather anomalies may be much more serious for winter-acclimatized animals. The low ability to downregulate the thermogenic machinery and maintenance of the capacity of heat production may lead to faster exhaustion of animal energy reserves. Climate data show that global changes correlate with climate unpredictability and increased probability of weather anomalies, also in winter (IPCC 2007). Phenotypic flexibility is considered as an important mechanism which may improve animal fitness in face of unpredictable changes of the environment (e.g., Canale and Henry 2010). Thus, if phenotypic flexibility of energetics interferes with photoperiodic control of energy metabolism and the seasonal signal overwhelms the phenotypic response to changes in thermal conditions, then the winter-acclimatized animals would be significantly constrained in their response to changing temperature. Our results indicate biologically significant interaction between short-term phenotypic flexibility and seasonal control for energy conservation showing that continued maintenance of metabolic rates in short days despite increase of *T*_a_s could prematurely deplete animal energy stores and eventually reduce their overwinter survival.

## Electronic supplementary material

Supplementary material 1 (PDF 231 kb)
