# Supplementary material for: Phenotypic flexibility of energetics in acclimated Siberian hamsters has a narrower scope in winter than in summer
Source: J Comp Physiol B. 2016 Jan 23;186:387–402. doi: 10.1007/s00360-016-0959-3 (PMC4791479; doi:10.1007/s00360-016-0959-3)
Supplement: Supplementary file 1 — Supplementary material 1 (PDF 231 kb) [file 360_2016_959_MOESM1_ESM.pdf]

Appendix 1.

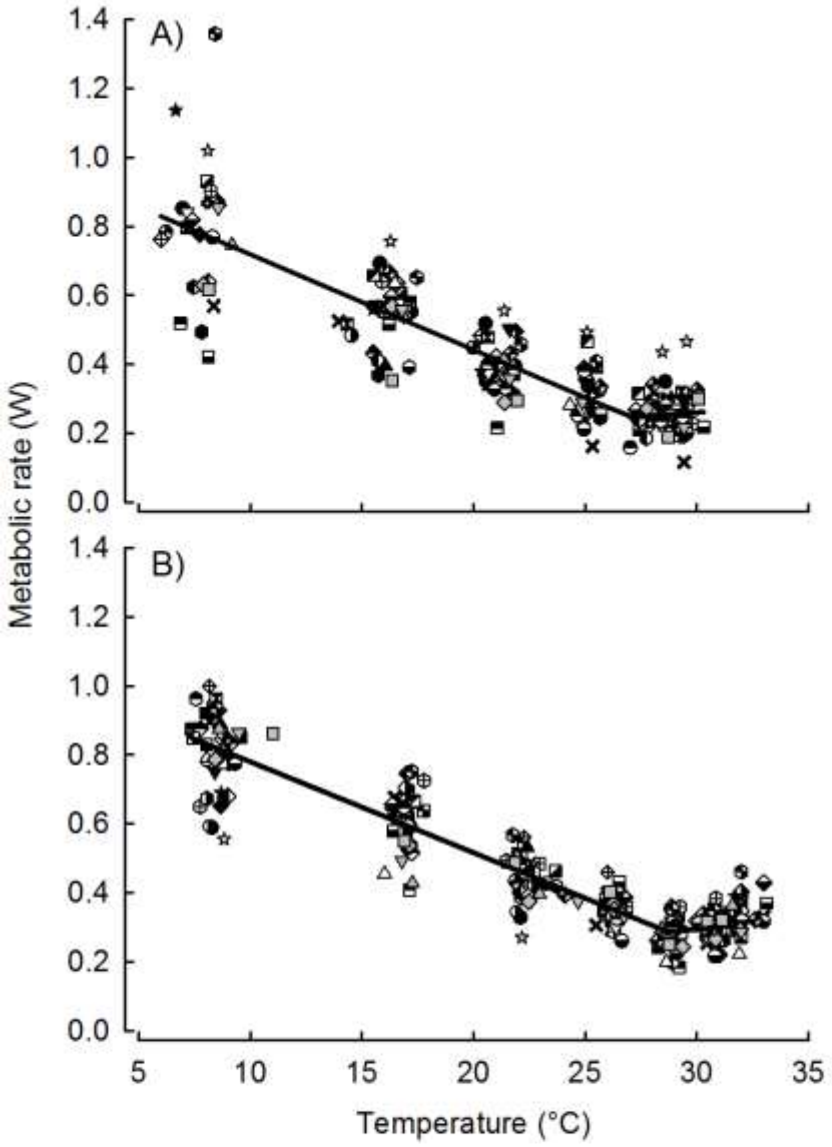

Figure S1. Metabolic rate as a function of ambient temperature (T_a) for Siberian hamsters *Phodopus sungorus* acclimated for 3 months to winter- (A) and summer-like conditions (B; see the text for the details of acclimation procedure). In winter we successfully collected data for 39, while in summer for 40 individuals. In winter lower critical temperature (T_{LC}) was at 26.9 ± 0.3°C, while in summer T_{LC} = 28.6 ± 0.2°C. The regression lines were fitted using least-squeres method, and for T_a's < T_{LC} they were described by the following equations: for winter – MR (W) = 1.00 -0.028 T_a (°C), r^2 = 0.66; for summer – MR (W) = 1.04 -0.026 T_a (°C), r^2 = 0.84. Note that each symbol represents different individual and that in each season one symbol indicates the same individual.

Table 1a. Body mass (g) of Siberian hamsters *Phodopus sungorus* randomly assigned to groups which were acclimated in different order to T_a's during winter and summer series of experiments. Body mass of individuals within group was compared using Friedman repeated measures test with Wilcoxon pairwise test for post-hoc comparisons. Values are mean ± SD, N – number of animals in each group, χ^2 – value of the test statistics, superscript letters indicate statistical differences at P<0.05.

Season	Order of acclimation T_a's (°C)	Acclimation Initial	First	Second	N	χ^2	P
Winter	10-10-20	30.15±3.46[a,b]	28.05±3.17[a,c]	26.05±2.57[b,c]	6	11.51	<0.001
	10-10-28	30.40±4.79[a]	28.56±3.85[a]	28.60±3.74	6	6.00	0.052
	10-20-10	32.49±3.29[a]	29.85±1.99[a]	29.92±1.69	7	3.50	0.192
	10-20-28	33.67±3.38	34.63±5.68	32.53±4.62	5	3.29	0.182
	10-28-10	32.88±2.52	31.29±2.93	29.73±3.56	5	7.12	0.024
	10-28-20	30.20±2.58[a,b]	27.69±1.62[a]	27.50±2.25[b]	6	8.91	0.005
Summer	20-10-20	39.60±4.16	40.95±4.78	40.34±3.38	7	3.50	0.192
	20-10-28	41.29±5.10	41.13±3.92	42.91±4.72	6	2.11	0.429
	20-20-10	38.38±1.25	39.64±2.18	40.34±1.70	5	4.42	0.124
	20-20-28	39.31±3.13	39.31±5.98	40.93±7.39	6	4.05	0.142
	20-28-10	41.35±1.06	40.60±1.59	41.16±1.29	5	0.96	0.691
	20-28-20	40.61±5.04	41.00±4.22	41.36±3.48	7	0.98	0.620

Table 2a. Basal metabolic rate (W) of Siberian hamsters *Phodopus sungorus* randomly assigned to groups which were acclimated in different order to T_a's during winter and summer series of experiments. Basal metabolic rate of individuals within each group was compared using Friedman repeated measures test with Wilcoxon pairwise test for post-hoc comparisons. Values are mean ± SD, N – number of animals in each group, χ^2 – value of the test statistics, superscript letters indicate statistical differences at P<0.05.

Season	Order of acclimation T_a's (°C)	Acclimation Initial	First	Second	N	χ^2	P
Winter	10-10-20	0.26±0.04[a]	0.21±0.04[a]	0.21±0.02	6	6.00	0.052
	10-10-28	0.27±0.05	0.25±0.04	0.22±0.05	6	4.05	0.142
	10-20-10	0.25±0.02	0.21±0.05	0.26±0.01	7	5.18	0.085
	10-20-28	0.26±0.03	0.24±0.04	0.24±0.03	5	0.96	0.691
	10-28-10	0.27±0.05	0.23±0.04	0.27±0.03	5	2.50	0.367
	10-28-20	0.25±0.02[a]	0.22±0.01[a]	0.24±0.02	6	6.00	0.052
Summer	20-10-20	0.31±0.04	0.33±0.05[a]	0.28±0.01[a]	7	4.34	0.112
	20-10-28	0.30±0.03	0.34±0.04	0.28±0.02	6	4.05	0.142
	20-20-10	0.30±0.01	0.28±0.02	0.38±0.05	5	4.81	0.093
	20-20-28	0.29±0.03[a]	0.30±0.04[b]	0.25±0.03[a,b]	6	8.91	0.006
	20-28-10	0.30±0.01	0.28±0.02	0.33±0.02	5	5.96	0.004
	20-28-20	0.30±0.04	0.30±0.02	0.30±0.03	7	0.14	0.964

Table 3a. Facultative nonshivering thermogenesis (W) of Siberian hamsters *Phodopus sungorus* randomly assigned to groups which were acclimated in different order to T_a's during winter and summer series of experiments. Body

masses within each order of acclimation were compared using Friedman repeated measures test with Wilcoxon pairwise test for post-hoc comparisons. Values are mean ± SD, N – number of animals in each group, χ^2 – value of the test statistics, superscript letters indicate statistical differences at P<0.05.

Season	Order of acclimation T_a's (°C)	Acclimation Initial	First	Second	N	χ^2	*P*
Winter	10-10-20	1.19 ± 0.03^{a}	$0.93\pm0.06^{a,b}$	1.20 ± 0.15^{b}	6	8.60	0.008
	10-10-28	$1.16\pm0.13^{a,b}$	$0.62\pm0.14^{a,c}$	$0.91\pm0.16^{b,c}$	6	11.51	<0.001
	10-20-10	1.30 ± 0.19^{a}	0.95 ± 0.19^{a}	1.27±0.23	7	7.42	0.021
	10-20-28	1.18±0.07	0.94±0.14	0.86±0.12	5	7.12	0.024
	10-28-10	1.28±0.04	0.45±0.12	1.12±0.36	5	7.12	0.024
	10-28-20	1.16 ± 0.11^{a}	0.58 ± 0.24^{a}	0.95±0.21	6	6.65	0.029
Summer	20-10-20	0.81 ± 0.27^{a}	$1.13\pm0.22^{a,b}$	0.88 ± 0.25^{b}	7	7.70	0.016
	20-10-28	$0.76\pm0.22^{a,b}$	$1.25\pm0.32^{a,c}$	$0.66\pm0.17^{b,c}$	6	11.51	<0.001
	20-20-10	0.82±0.19	0.68±0.18	1.07±0.18	5	4.81	0.093
	20-20-28	0.74 ± 0.15^{a}	0.78±0.31	0.51 ± 0.07^{a}	6	4.05	0.142
	20-28-10	0.77±0.19	0.63±0.20	1.04±0.27	5	5.96	0.004
	20-28-20	0.82 ± 0.26^{a}	0.58 ± 0.21^{a}	0.69±0.27	7	5.74	0.051

Table 4a. Minimum thermal conductance (mW°C^{-1}cm^{-2}) of Siberian hamsters *Phodopus sungorus* randomly assigned to groups which were acclimated in different order to T_a's during winter and summer series of experiments. Minimal thermal conductance of individuals within each group was compared using Friedman repeated measures test with Wilcoxon pairwise test for post-hoc comparisons. Values are mean ± SD, N – number of animals in each group, χ^2 – value of the test statistics, superscript letters indicate statistical differences at P<0.05.

Season	Order of acclimation T_a's (°C)	Acclimation Initial	First	Second	N	χ^2	*P*
Winter	10-10-20	0.25±0.07	0.24±0.04	0.22±0.10	6	2.11	0.429
	10-10-28	0.25±0.06	0.27±0.02	0.32±0.12	6	2.11	0.429
	10-20-10	0.27±0.04	0.24±0.09	0.26±0.05	7	0.14	0.964
	10-20-28	0.26±0.04	0.31±0.07	0.26±0.09	5	0.19	0.954
	10-28-10	0.27±0.03	0.21±0.09	0.23±0.05	5	0.19	0.954
	10-28-20	0.22±0.07	0.24±0.04	0.22±0.08	6	0.81	0.740
Summer	20-10-20	0.24±0.05	0.28±0.04	0.27±0.04	7	0.70	0.768
	20-10-28	0.26±0.03	0.26±0.02	0.26±0.04	6	2.11	0.429
	20-20-10	0.25±0.04	0.24±0.03	0.26±0.03	5	0.19	0.954
	20-20-28	0.25±0.03	0.24±0.02	0.21±0.04	6	2.76	0.252
	20-28-10	0.25±0.02	0.25±0.03	0.27±0.05	5	0.19	0.954
	20-28-20	0.26±0.05	0.25±0.03	0.25±0.03	7	0.70	0.768
